# Huntington's Disease Induced Cardiac Amyloidosis Is Reversed by Modulating Protein Folding and Oxidative Stress Pathways in the *Drosophila* Heart

**DOI:** 10.1371/journal.pgen.1004024

**Published:** 2013-12-19

**Authors:** Girish C. Melkani, Adriana S. Trujillo, Raul Ramos, Rolf Bodmer, Sanford I. Bernstein, Karen Ocorr

**Affiliations:** 1 Department of Biology, Molecular Biology and Heart Institutes, San Diego State University, San Diego, California, United States of America; 2 Development and Aging Program, Sanford-Burnham Institute for Medical Research, La Jolla, California, United States of America; Stanford University School of Medicine, United States of America

## Abstract

Amyloid-like inclusions have been associated with Huntington's disease (HD), which is caused by expanded polyglutamine repeats in the Huntingtin protein. HD patients exhibit a high incidence of cardiovascular events, presumably as a result of accumulation of toxic amyloid-like inclusions. We have generated a *Drosophila* model of cardiac amyloidosis that exhibits accumulation of PolyQ aggregates and oxidative stress in myocardial cells, upon heart-specific expression of Huntingtin protein fragments (Htt-PolyQ) with disease-causing poly-glutamine repeats (PolyQ-46, PolyQ-72, and PolyQ-102). Cardiac expression of GFP-tagged Htt-PolyQs resulted in PolyQ length-dependent functional defects that included increased incidence of arrhythmias and extreme cardiac dilation, accompanied by a significant decrease in contractility. Structural and ultrastructural analysis of the myocardial cells revealed reduced myofibrillar content, myofibrillar disorganization, mitochondrial defects and the presence of PolyQ-GFP positive aggregates. Cardiac-specific expression of disease causing Poly-Q also shortens lifespan of flies dramatically. To further confirm the involvement of oxidative stress or protein unfolding and to understand the mechanism of PolyQ induced cardiomyopathy, we co-expressed expanded PolyQ-72 with the antioxidant superoxide dismutase (SOD) or the myosin chaperone UNC-45. Co-expression of SOD suppressed PolyQ-72 induced mitochondrial defects and partially suppressed aggregation as well as myofibrillar disorganization. However, co-expression of UNC-45 dramatically suppressed PolyQ-72 induced aggregation and partially suppressed myofibrillar disorganization. Moreover, co-expression of both UNC-45 and SOD more efficiently suppressed GFP-positive aggregates, myofibrillar disorganization and physiological cardiac defects induced by PolyQ-72 than did either treatment alone. Our results demonstrate that mutant-PolyQ induces aggregates, disrupts the sarcomeric organization of contractile proteins, leads to mitochondrial dysfunction and increases oxidative stress in cardiomyocytes leading to abnormal cardiac function. We conclude that modulation of both protein unfolding and oxidative stress pathways in the *Drosophila* heart model can ameliorate the detrimental PolyQ effects, thus providing unique insights into the genetic mechanisms underlying amyloid-induced cardiac failure in HD patients.

## Author Summary

Huntington's disease (HD) is associated with amyloid-like inclusions in the brain and heart, and accumulation of amyloid protein is associated with neurodegeneration and cardiomyopathy. Recent studies suggest that HD patients show increased susceptibility to cardiac failure. However, the mechanisms by which disease-causing poly-glutamine repeats (PolyQ) cause heart dysfunction in these patients are unclear. We have developed a novel *Drosophila* heart model that exhibits significant GFP-positive aggregates upon HD-causing PolyQ expression in myocardial cells resulting in PolyQ length-dependent physiological defects. Modulation of protein folding and oxidative stress pathways in this system reduced the number of aggregates and reversed the cardiac dysfunction in response to expression of disease-causing PolyQ. The ability to explore PolyQ-associated mechanisms of cardiomyopathy in a genetically tractable whole organism, *Drosophila melanogaster*, promises to provide novel insights into the relationship between amyloid accumulation and heart dysfunction. Our findings not only impact the understanding of PolyQ-induced cardiomyopathy but also other human cardiac diseases associated with oxidative stress, mitochondrial defects and protein homeostasis.

## Introduction

Amyloidosis constitutes a large group of diseases characterized by the misfolding of proteins and the accumulation of protein aggregates in different tissues [1]–[3]. Huntington's disease (HD) is an inherited neurodegenerative disorder caused by mutations in the Huntingtin (HTT) protein which result in expanded Poly-glutamine (PolyQ, CAG_n_) repeats that cause aggregation-prone amyloidosis [4]–[7]. The molecular mechanism that leads to HD is not fully understood and presently no effective treatment exists [4], [8]–[10]. It has been well established that the length of the PolyQ repeat is important in the progression of disease [4], [5]. HTT with 6–35 PolyQ repeats does not cause HD. However, HTT with more than 40 PolyQ (CAG_40_) repeats results in HD [4], [5], [11]. In general HD is primarily considered as an aggregation-based disease; however, some studies have shown that disease-causing PolyQ repeats in HTT make it prone to misfolding and aggregation [4]–[7], [12]–[15].

HTT is expressed in several tissues in addition to the brain, including heart and skeletal muscles [11], [16]–[18] and is known to be involved in protein trafficking, vesicle transport and transcriptional events [4], [11]. HD is also associated with skeletal muscle atrophy [11], [19] and multiple epidemiological studies have shown that cardiovascular diseases and cardiac failure are the second leading cause of mortality in HD patients [8], [18], [20]. Cardiac failure is implicated as the cause of death in over 30% of HD patients, compared to 2% of the age-matched non–HD patients [8]–[11], [18], [20]. Although, the mechanism whereby mutant HTT causes muscle atrophy and cardiac defects is not known, it is possible that an increase in protein misfolding and the consequent high energy burden in cardiac cells play roles [8], [10], [11], [16], [18]. In support of this, recent evidence demonstrates nuclear and cytoplasmic PolyQ aggregates in non-CNS tissue [11], [16], [18]. Furthermore, neuronal expression of mutant HTT protein with expanded PolyQ or cardiac-specific expression of only the PolyQ pre-amyloid oligomers in mice leads to cardiac defects [10], [21], [22]. Furthermore, expression of mutant PolyQ-81 in mice and in rat neonatal cardiomyocytes results in amyloid as well as PolyQ-positive aggregates in the cytoplasm and over-expression of a chaperone αB-crystallin reduces PolyQ-induced aggresomes [21], [23]. Moreover, reduction of aggresomes upon over-expression αB-crystallin results in higher levels of amyloid oligomer and enhances toxicity [23]. Despite the availability of cell and mouse models to examine/investigate PolyQ expression in the heart, little is known about the mechanism that leads to cardiac dysfunction.

We have previously established the *Drosophila* heart as a useful model system to generate insights into the genetic basis of heart development and to elucidate the genetic interactions underlying heart physiology and age-dependent deterioration [24]–[27]. Recently, we showed in this genetic model that knock-down of the chaperone UNC-45 significantly reduced myosin expression and led to severe cardiac dilation [28]. Protein folding and oxidative stress pathways have previously been implicated in the development/pathology of HD [5], [6], [10], [18], [19], however, their involvement with cardiac phenotypes has not been explored. In the current study, we manipulate these two pathways to attempt to suppress the cardiac defects induced by mutant HD-associated PolyQ repeat lengths. These defects include the accumulation of GFP-positive aggregates, mitochondrial defects, oxidative stress and both functional and morphological cardiac abnormalities. We also found that cardiac over-expression (OE) of the chaperone UNC-45 suppressed amyloid deposition and ameliorated the heart function defects to some extent. In addition, OE of SOD as well as feeding with the dietary antioxidant resveratrol also partially suppressed the amyloid-induced cardiac dysfunction, whereas hydrogen peroxide feeding aggravated the heart defects. Our data suggest that the protein folding and ROS pathways interact in mediating the effects of mutant HTT as a near complete reversal of the cardiac defects was achieved when both pathways were modulated simultaneously in flies expressing disease-causing PolyQ repeats. Thus, our data show a deleterious effect of mutant PolyQ aggregates on cardiac function and indicate that these effects are the result of protein misfolding and/or concomitant oxidative stress.

## Results

### Expression of disease causing PolyQ in the heart causes cardiac dilation and reduced contractility

**Figure 1:**
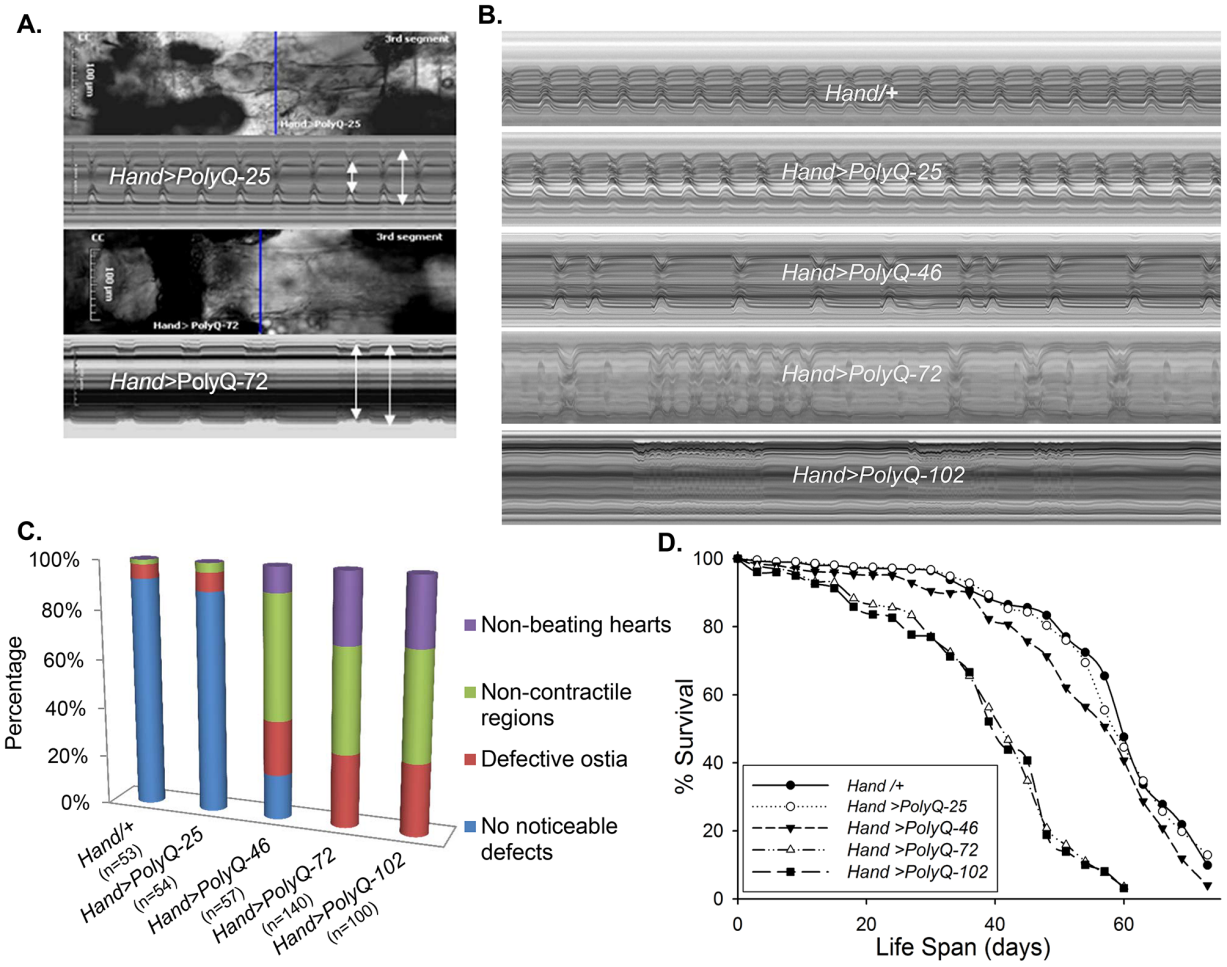
Cardiac defects are associated with expression of mutant Poly-Q. (**A**) Images of semi-intact fly heart preparations during systole and corresponding M-mode records obtained from high-speed movies of 1 week old control (*PolyQ-25*, top and mutant *PolyQ-72*, bottom) hearts. M-mode records (6 sec) show heart wall movements (at the location indicated by the blue line) over time [72]. Double-headed arrows in the M-mode records indicate the location of the heart walls and the heart diameter during diastole and systole. Compared to PolyQ-25, hearts expressing PolyQ-72 showed significant dilation in the conical chamber and in the third abdominal segment of the cardiac tube. Cardiac arrhythmias are also apparent in the M-mode record from the PolyQ-72 expressing heart. (**B**) M-mode records from 3-week old control (*Hand/+*, *PolyQ-25*) and mutant (*PolyQ-46, PolyQ-72, PolyQ-102*) flies. Compared to *Hand/+* or *PolyQ-25*, cardiac-specific expression of *PolyQ-46*, *PolyQ-72* and *PolyQ-102* showed increasingly arrhythmic beating patterns. Expression of *PolyQ-72* and *PolyQ-102* show frequent asystolic (non-beating) events. (**C**) Summary of the qualitative cardiac defects from 3-week old control (*Hand/+*, *PolyQ-25*) and mutant (*PolyQ-46, PolyQ-72, PolyQ-102*) flies showing the percent of flies exhibiting defective ostia, one or more non-contractile regions, and non-beating hearts. (**D**) Cardiac-specific expression of mutant *PolyQ-72* and *PolyQ-102* resulted in a significant reduction in lifespan compared to flies expressing non-disease causing *PolyQ-25* and wild-type *Hand*/+ controls (p<0.001). Cardiac-specific expression of PolyQ-46 caused a small but significant decrease in lifespan (p<0.05). Graph plots % survival (n = 150–200 for each group) vs. time post-eclosion.

To evaluate cardiac function following PolyQ-induced cardiomyopathy, we obtained *Drosophila* transgenic lines [5] expressing enhanced-GFP-tagged control or mutant Htt fragments (UAS-Httex1-QneGFP) with different PolyQ repeat lengths (Q25, Q46, Q72, and Q103). For simplicity, Httex1-Q25-eGFP, Httex1-Q46-eGFP, Httex1-Q72-eGFP and Httex1-Q102-eGFP are referred to as PolyQ-25, PolyQ-46, PolyQ-72 and PolyQ-102, respectively. Using the heart-specific driver *Hand*, we observed severe cardiac defects and/or extreme dilation upon expression of disease-causing PolyQ-46, PolyQ-72 and PolyQ-102, whereas the shorter PolyQ-25 had no measurable effect. Figure 1A shows contracted hearts from 1-week old flies with cardiac-specific expression of PolyQ-72 and an age-matched control expressing PolyQ-25. The heart tube expressing PolyQ-72 is clearly much less contracted during systole than is the PolyQ-25 control. Heart wall diameters during both systole and diastole are indicated by double headed arrows in the M-mode records produced from high speed movies; the line of pixels used to produce these records is indicated by the blue line in the still image. The ability of the PolyQ-72 heart to contract during systole is much reduced compared to the control heart. A comparison of representative M-mode records for 3-week old flies from each of the transgenic lines and wild type controls is shown in Figure 1B and demonstrates a progressive increase in cardiac arrhythmia with increased length of PolyQ. A comparison of PolyQ-25, PolyQ-46 and PolyQ-72 heart contractility, dilation and arrhythmia is also shown in supplementary movie S1. Hearts expressing the longer repeats, PolyQ-72 and PolyQ-102, also showed significant cardiac dilation in addition to arrhythmia (Figure 1B). Hearts from flies with cardiac-specific expression of mutant PolyQ also exhibited additional functional and morphological defects including floppy, non-contractile ostia and one or more non-contractile myocardial cells, primarily in the conical chamber (CC) and the adjacent chamber (Figure 1C). In PolyQ-72 and PolyQ-102 expressing hearts there were frequent asystolic periods as well as hearts that were completely unable to beat. The incidence of these qualitative defects is quantified in Figure 1C. In addition to these heart function defects, cardiac specific expression of the two longer PolyQ proteins (PolyQ-72 and PolyQ-102) significantly shortens the lifespan of flies (Fig. 1 D and Table S1).

**Figure 2:**
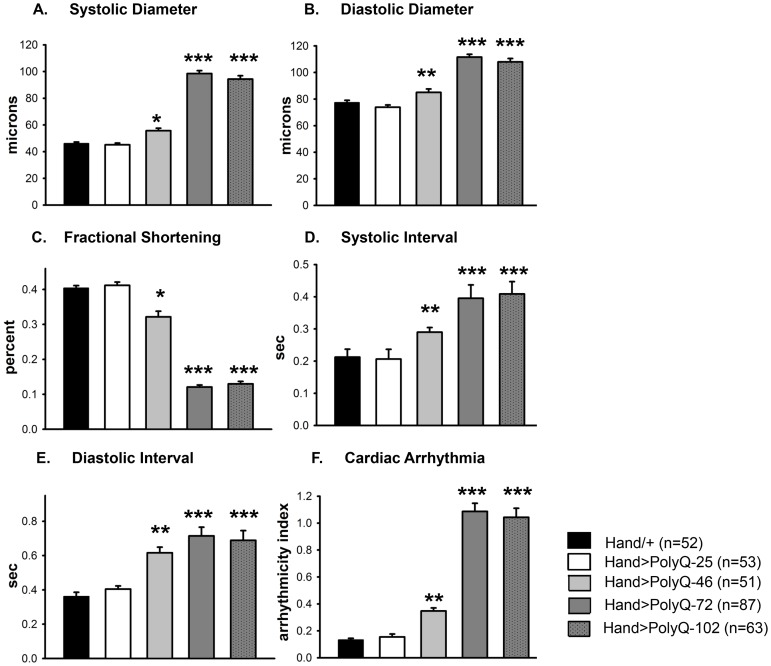
Disease-causing Poly-Q yields cardiac defects in 3 weeks. (**A**) Systolic and (**B**) diastolic diameters of the hearts from *PolyQ-46, PolyQ-72 and PolyQ-102* expressing flies were significantly higher than those of age-matched controls (*Hand*/+ or *PolyQ-25*). (**C**) Cardiac contractility was quantified as % Fractional Shortening; hearts expressing *PolyQ-46*, *PolyQ-72* and *PolyQ-102* showed significantly reduced contractility compared to control hearts. (**D**) Systolic intervals were prolonged in hearts expressing *PolyQ-46*, *PolyQ-72* and *PolyQ-102* as well as (**E**) diastolic intervals compared to control hearts (*Hand*/+ or *PolyQ-25*). (**F**) Cardiac arrhythmicity (AI) was significantly increased in hearts expressing *PolyQ-46*, *PolyQ-72* and *PolyQ-102* compared to controls. There was no statistical difference between *Hand*/+ and *PolyQ-25* hearts in any of the cardiac function parameters. All data are shown as means ± SE; statistical significance was determined using one-way ANOVA and Dunnett's post hoc test. Significant differences were assumed for p<0.05. (* = p<0.05, ** = p<0.01, *** = p<0.001).

Quantification of functional parameters from the high-speed movies demonstrated a significant increase in diameters during both systole (Figure 2A) and diastole (Figure 2B) compared to hearts expressing PolyQ-25 and wild-type controls; this dilation was more severe for the longer PolyQ repeats (PolyQ-72 and PolyQ-102). The observed cardiac dilation was accompanied by a significant reduction in heart contractility, measured as a decreased fractional shortening (% FS) in the PolyQ-46, PolyQ-72 and PolyQ-102 expressing hearts (Figure 2C). Long PolyQ repeats induced significant increases in both the systolic and diastolic intervals and this effect again appeared to be dependent on the “dose” of PolyQ (Figure 2D, 2E). The incidence in cardiac arrhythmias was also quantified (arrhythmia index) [27]–[29], and showed a PolyQ dose-dependent increase (Figure 2F, see also Figure 1B). Cardiac specific expression of PolyQ-25 did not significantly alter any of the measured cardiac function parameters when compared to hearts from control flies that lacked any PolyQ expression (*Hand-Gal4/+*). Similar alterations in cardiac physiological parameters were observed in response to disease-causing PolyQ expression (PolyQ-46, PolyQ-72 and PolyQ-102) in younger, 1 week old flies (Figure S1A to S1F). Taken together our data indicate that all the cardiac defects we observe are PolyQ-length dependent and suggest that the PolyQ-72 repeat length is sufficient to exert the maximal deleterious effect on these hearts.

### Cardiac expression of disease-causing PolyQ causes accumulation of aggregates and myofibrillar defects

**Figure 3:**
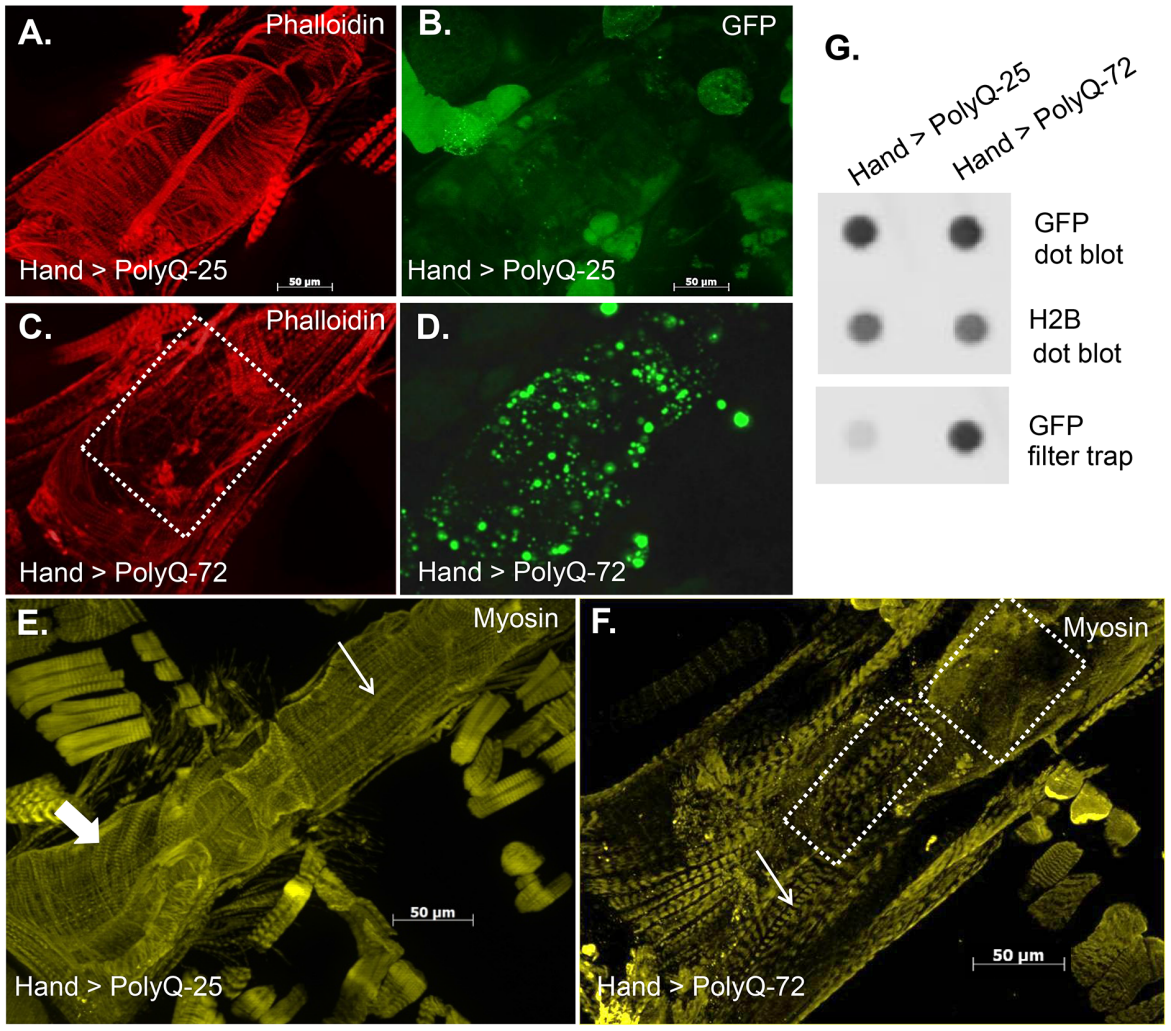
Mutant PolyQ causes structural defects and GFP-positive aggregates in the fly heart. (**A**) Hearts from 3-week old PolyQ-25 controls show typical circumferential arrangements of actin-containing myofibrils within the cardiomyocytes and (**B**) homogeneously distributed GFP in the cardiomyocytes. (**C**) In hearts expressing *PolyQ-72* the myofibrillar organization is severely disrupted (region indicated by dashed lines) with a loss of actin-containing myofibrils. (**D**) GFP-positive aggregates of various sizes are distributed throughout the cardiomyocytes in hearts expressing PolyQ-72. (**E**) Heart from 3-week old fly expressing *PolyQ-25* shows myosin-containing myofibrils. This control heart shows typical circumferential arrangement of myosin-containing myofibrils (thick arrow). (**F**) The circumferential arrangement of myosin-containing myofibrils is nearly lost upon expression of mutant *PolyQ-72* (F, dashed boxes). Thin arrows in E and F indicate myofibrils in the non-cardiac longitudinal fibers. (**G**). Dot blot protein assay (top) showing similar overall expression of Htt-PolyQ-25 GFP (control) and Htt-PolyQ-72 GFP protein relative to histone H2B (middle, loading control). Filter trap assay (bottom) shows more GFP positive protein aggregates present in the Htt-PolyQ-72 GFP expressing hearts compared to control hearts.

To explore whether the cardiac physiological dysfunction in response to cardiac-specific expression of PolyQ is the result of amyloid accumulation we used a Green Fluorescent Protein (GFP) tag to visualize PolyQ proteins and amyloid deposits and phalloidin to detect F-actin, revealing the myofibrillar organization within myocardial cells. Hearts expressing non-disease causing PolyQ-25 (control) show densely packed actin-containing myofibrils arranged in a circumferential pattern within the cardiomyocytes (Figure 3A) and GFP-tagged PolyQ was found to be distributed homogeneously throughout the cytoplasm (Figure 3B). In contrast, expression of disease-causing PolyQ-72 resulted in noticeably reduced myofibrillar content and in severe myofibrillar disorganization (Figure 3C, dashed box). Interestingly, we also observed the presence of many GFP-positive aggregates of various sizes throughout the cardiomyocytes (Figure 3D). We used a well-documented filter trap assay [30]–[32] to confirm this increase in aggregate formation upon expression of mutant PolyQ-72. Although expression of both control PolyQ-25 and mutant PolyQ-72 protein was virtually the same relative to histone H2B (Fig. 3G, top and middle panels), the heart-specific expression of mutant PolyQ-72 resulted in a significant increase in GFP-positive aggregates compared to control hearts (Figure 3G, bottom panel).

We also used antibody against muscle myosin to explore the effect of mutant PolyQ on the myosin organization within the myofibrils. Myosin organization in myofibrils from control hearts exhibits a similar circumferential arrangement as for F-actin (thick arrow in Figure 3E); however, the myosin pattern appears significantly aberrant upon expression of PolyQ-72 (Figure 3F). In fact the majority of the staining visible in Fig. 3F is due to myosin in the non-cardiac longitudinal muscle fibers that run ventrally along the cardiac tube (thin arrows in Figure 3E and 3F). Some disorganized myosin-containing myofibrils are still seen upon expression of mutant PolyQ (Figure 3F, dashed boxes). This phenotype is reminiscent of knock-down of the myosin-specific chaperone UNC-45 [28], suggesting that long PolyQ aggregates might interfere with chaperone function. These data indicate that the presence of toxic aggregates leads to a reduction in cardiac myosin-actin content with disorganized myofibrils.

### Ultrastructural analysis revels mitochondrial and myofibrillar defects upon expression of mutant PolyQ

**Figure 4:**
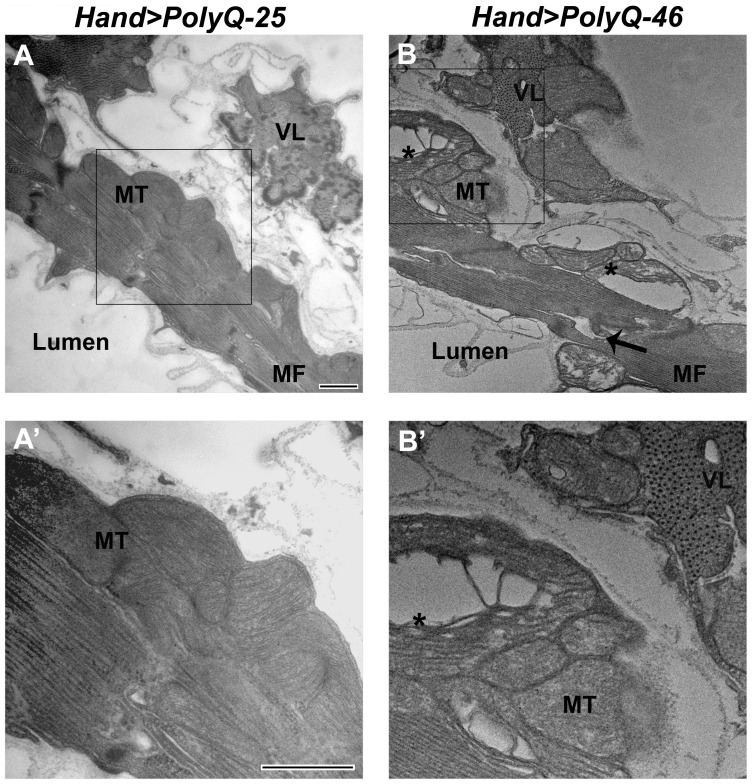
Mutant PolyQ causes myofibrillar and mitochondrial ultrastructural defects in the fly heart. (A, A') Electron micrograph of a cross section through the dorsal vessel of 4-week old PolyQ-25 controls reveals a layer of contractile myocardial cells which form the heart tube and a non-contractile supporting layer of ventral-longitudinal fibers (VL). Note that myofibrils (MF) are intact and mitochondria (MT) contain densely packed cristae. (B, B') In contrast, the myocardial layer of 4-week old PolyQ-46 hearts contains gaps indicating some myofibrillar degeneration (arrow) and severely fragmented mitochondria (asterisks). Bars, 500 nm.

The ultrastructure of *Drosophila* cardiac muscle has been described previously in detail by Lehmacher et al. [33]. Transmission electron micrographs of transverse sections of 4 week-old hearts from PolyQ-25 control flies reveal a layer of contractile cardiomyocytes and a supporting layer of non-cardiac ventral-longitudinal fibers (VL, Figure 4A). Myocardial cells from PQ-25 controls contain mitochondria with densely packed cristae (4A, MT) that can be seen adjacent to the myofibrils (Figure 4A, MF). In contrast, micrographs from PolyQ-46 hearts show evidence of myofibrillar degeneration (4B, arrow) and severe mitochondrial fragmentation (4B, B', asterisks). Such mitochondrial fragmentation and alterations in cristae structure have previously been linked to increased apoptotic activity in primary striatal cultures from YAC128 HD transgenic mice as well as in neuronal expression of mutant HTT protein with expanded PolyQ in a mouse heart model [10], [34]. These defects are even more severe in hearts expressing longer forms of PolyQ-72 with near complete loss of myofibrillar architecture.

We also looked for autophagosome/lysosome structures and observed a significant amount of LysoTracker positive punctae upon expression of mutant PolyQ-72 (Figure S2 E). Significantly, most of the staining is co-localized with PolyQ-GFP punctae (Figure S2 F). In contrast, almost no GFP- or LysoTracker-positive punctae are seen upon expression of non-disease causing PolyQ-25 (Figure S2 A–C) suggesting a direct link between expression of mutant PolyQ and activation of the autophagy pathway.

### Oxidative stress plays a role in PolyQ-induced cardiac defects

**Figure 5:**
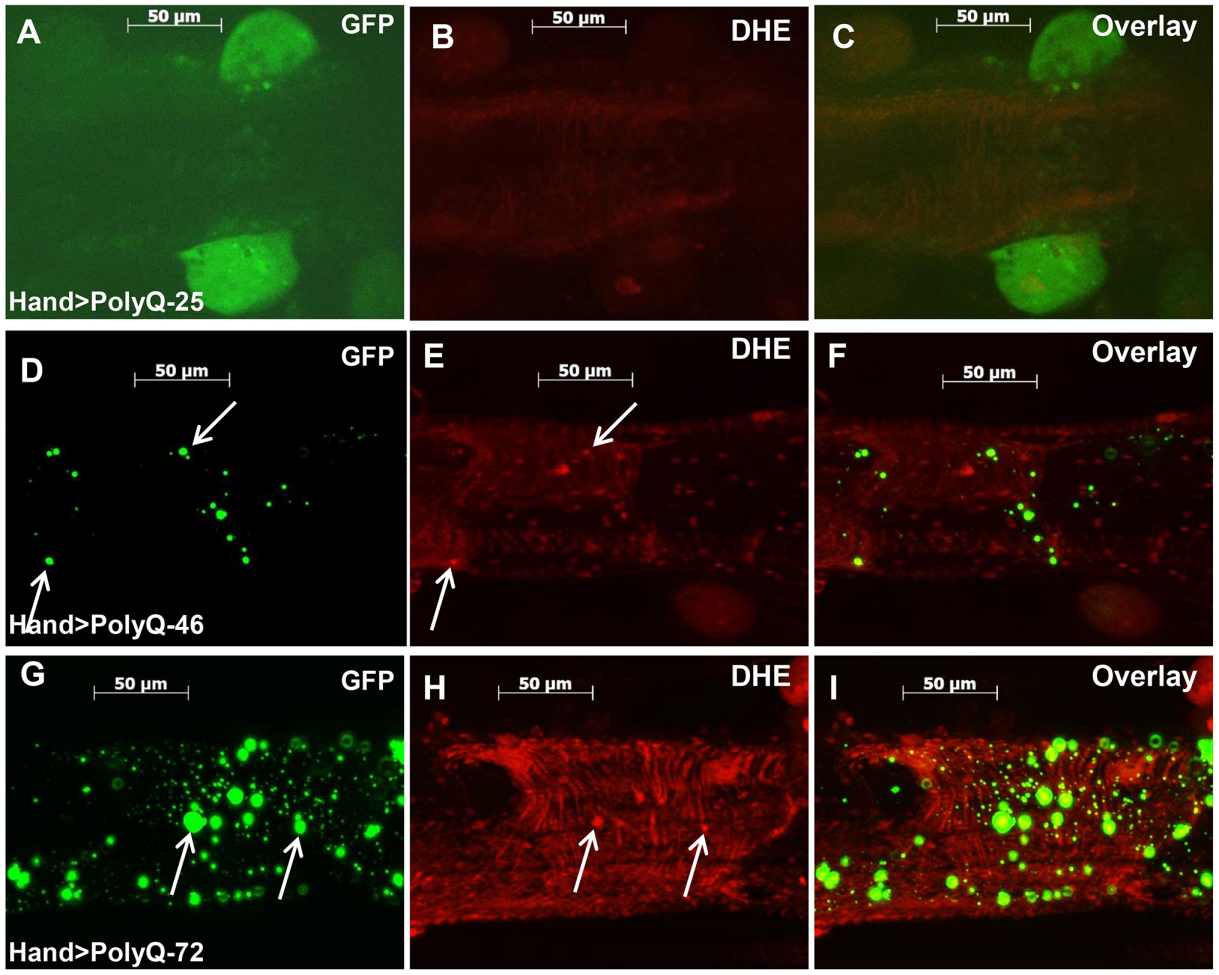
Mutant PolyQ induces oxidative stress. (**A–C**) Immunofluorescence micrographs showing GFP expression and DHE staining in hearts expressing PolyQ-25. (**D–F**) GFP expression and DHE staining in hearts expressing PolyQ-46 and (**G–I**) GFP expression and DHE staining in hearts expressing PolyQ-72. Expression of mutant PolyQ correlates with enhanced DHE staining and more aggregation compared to age-matched control (PolyQ-25), with moderate length PolyQ (PolyQ-46) expressing hearts showing less staining than PolyQ-72 hearts. Note that many of the GFP-positive aggregates co-localize with areas of strong DHE staining in the merged images for both PolyQ-46 and 72 (arrows).

The mitochondrial defects, myofibrillar disorganization and cardiac function abnormalities observed upon expression of mutant PolyQ could arise from aggregate-induced oxidative stress. We used dihydroethidium (DHE) to evaluate the role of reactive oxygen species (ROS) production in mediating the effects of disease-causing PolyQ. Cardiac expression of PolyQ-46 and PolyQ-72 resulted in 2- and 5-fold increases in DHE staining respectively (Figure 5E and 5H) compared to age-matched PolyQ-25 control hearts (Figure 5B). Furthermore, a number of the mutant-PolyQ induced GFP-aggregates colocalize with areas of strong DHE staining (arrows in Figure 5D and E; and Figure 5G and H) while expression of PolyQ-25 shows almost no GFP-positive punctae or DHE staining. These results confirm an association between PolyQ-induced aggregates and oxidative stress.

**Figure 6:**
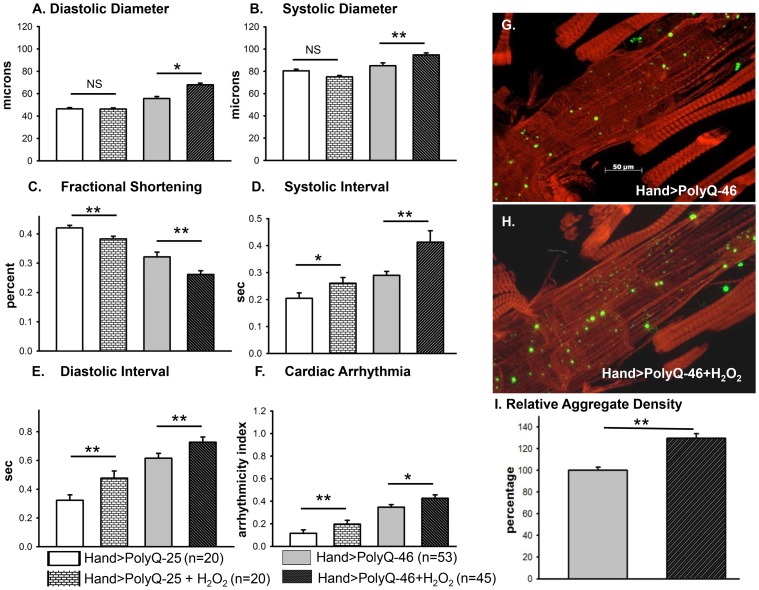
Cardiac function of hearts expressing control PolyQ-25 and mutant PolyQ-46 affected differently by oxidant. (**A & B**) The already large systolic and diastolic heart diameters were further increased in the presence of the oxidant H_2_O_2_ in the hearts expressing PolyQ-46. (**C**) Cardiac contractility (% FS) was further decreased in the presence of the oxidant in hearts expressing PolyQ-46. (**D & E**) The already prolonged systolic and diastolic intervals in hearts expressing PolyQ-46 were further increased by feeding flies the oxidant H_2_O_2_. (**F**) The incidence of cardiac arrhythmias was further increased in the presence of oxidant compared to age-matched PolyQ-46 expressing hearts without oxidant (A–F). Hearts expressing PolyQ-25 in the presence of oxidant showed cardiac defects without cardiac dilations (A–F). Data are shown as means ± SE. (**G**) Actin staining and GFP aggregates in heart expressing PolyQ-46 without oxidant. (**H**) PolyQ-46 expressing heart from fly fed oxidant showed more myofibrillar defects and contained more GFP-positive aggregates compared to controls. (**I**) Number of aggregates/unit area is expressed as percentage relative to control. Flies fed oxidant had 30% more aggregates than did hearts from age-matched PolyQ-46 without oxidant. For all data statistical significance was determined using one-way ANOVA and Dennett's post-hoc test. Significant differences were assumed for p<0.05. (* = p<0.05, ** = p<0.01, *** = p<0.001).

To explore whether induction of oxidative-stress could aggravate the PolyQ phenotype, flies expressing PolyQ-25 and PolyQ-46 in cardiac tissue were fed H_2_O_2_ for 3-weeks during adulthood. PolyQ-46 expressing hearts in the presence of oxidant showed significantly increased cardiac dilation. However, no such enlargements of cardiac diameters were seen in PolyQ-25 expressing hearts in the presence of oxidant (Figure 6A–6B). Although fractional shortening was decreased and cardiac arrhythmias were increased in PolyQ-46 expressing hearts by H_2_O_2_ feeding, both were affected to a similar extent as were control PolyQ-25 expressing hearts (Fig. 6C–F). Feeding oxidant to non-PolyQ expressing wild-type flies (*Hand-Gal4/+*) had similar minimal effects on cardiac parameters as for the PolyQ-25 controls (Figure S3A–S3F). The increased incidence of arrhythmia and reduced contractility of hearts expressing PolyQ-72 was also aggravated in the presence of oxidant (Figure S4A, S4B).

While PolyQ-46 hearts in the absence of oxidant do exhibit sparsely distributed amyloid-aggregates, the presence of H_2_O_2_ results in an increase (30%) in the density of aggregates (green GFP-punctae, Figure 6G, 6I). Furthermore, treatment with oxidant also resulted in more myofibrillar disorganization and loss compared to age-matched PolyQ-46 without oxidant (compare Figures 6G and 6H). Interestingly, muscle fiber organization remained virtually unchanged in hearts from wild-type controls (*Hand/+*) and PolyQ-25 expressing controls when fed H_2_O_2_ (compare Figure S3G with S3H and S3I with S3J). These data support the idea that oxidative stress enhances the accumulation of mutant PolyQ aggregates (GFP-punctae), myofibrillar disorganization and loss of actin-containing myofibrils and that these aggregates contribute to the cardiac dilation and heart function defects we observe. Additionally, our results show that oxidative stress exerts differential effects on heart function and structure depending upon the presence or absence of PolyQ aggregates. It is also possible that treatments with H_2_O_2_ may lead to secondary stress (such as initiation of the heat shock program). Assuming secondary stresses in response to H_2_O_2_ were similar between these two groups of flies, the only explanation for these more severe defects in the PolyQ-46 expressing group is an interaction between ROS signaling and mutant PolyQ expression. The H_2_O_2_ treatments may also affect other tissues such as neuronal tissue; therefore, it will be interesting to determine if control and mutant PolyQ affect cardiac and neuronal tissue under oxidative stress in a similar manner.

### Transgenic over-expression of SODs rescues the Poly-Q induced cardiac mitochondrial and functional defects

**Figure 7:**
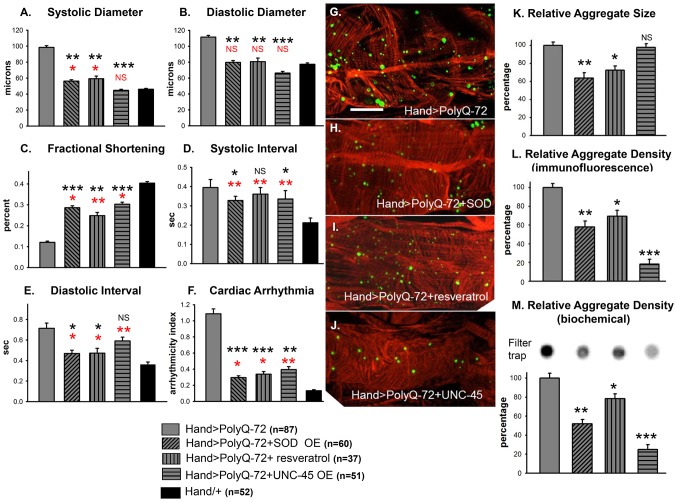
Over-expression of SOD or treatment with resveratrol or over-expression of UNC-45 all rescue PolyQ-72 induced cardiac dysfunction to some extent. (**A**) PolyQ-72 induced cardiac systolic and (**B**) diastolic diameters are reduced nearly to wild-type (*Hand*/+) levels. (**C**) Cardiac contractility (% FS) is improved upon over-expression of SOD, treatments with resveratrol or over-expression of UNC-45. (**D**) Systolic and (**E**) diastolic intervals and (**F**) cardiac arrhythmias from hearts expressing PolyQ-72 and expressing SOD-1 or fed resveratrol or expressing UNC-45 were reduced approaching towards wild-type values compared to hearts expressing PolyQ-72 alone. Hearts were from 3-week old flies. (A–F) Data shown as mean ± SE. (**G**) Myofibrillar organization and GFP-positive aggregates in PolyQ-72 expressing hearts and (**H**) in PolyQ-72 hearts over-expressing SOD or (**I**) flies fed resveratrol or (**J**) hearts over-expressing UNC-45. (**K**) Relative GFP-aggregate size was reduced in response to SOD over-expression or feeding resveratrol, however, over-expression of UNC-45 did not affect the average aggregate size. (**L**) Quantification of the number of aggregates per unit area shows that all treatments significantly reduced the density of aggregates formed in response to PolyQ-72 expression. K and L shows averaged data from 4 to 6 hearts, expressed as a percent relative to the mutant PolyQ. (**M**) Quantification of relative GFP-aggregate density with the filter trap assay. An example of one filter trap blot is shown (top), and the averaged densitometric scan results expressed as a percent relative to the mutant PolyQ (bottom). Compared to Htt-PolyQ-72, treatments with resveratrol or over-expression of SOD or UNC-45 reduced PolyQ-induced aggregation to different extents. For all panels significance was determined using a one-way ANOVA and Dunnett's post hoc test. Significant differences were assumed for p<0.05. (*** = p<0.001, ** = p<0.01, * = p<0.05, NS = no statistical difference, p>0.05). Red * and NS indicate differences relative to the *Hand*/+ control; black text indicates differences relative to mutant PolyQ-72. Scale bar in G is 20 µm.

Because mutant PolyQ expressing hearts exhibited oxidative stress, mitochondrial defects as well as aggravated cardiac defects in response to hydrogen peroxide feeding, we tested whether over-expression of superoxide dismutase (SOD) could rescue the PolyQ-induced cardiomyopathy. We over-expressed SOD-1 or SOD-2 along with PolyQ-72 in fly hearts and examined the effect on cardiac function (Figure 7A–7F). In hearts from 3-week old flies over-expressing SOD-1 along with PolyQ-72 the cardiac dilation was significantly reduced compared to hearts expressing PolyQ-72 alone and was nearly the same as for wild-type *Hand/+* hearts (Figure 7A, 7B). Cardiac contractility was also improved in SOD overexpressing hearts (Figure 7C). Both diastolic and systolic intervals were significantly lower (Figure 7D, 7E) and the incidence of arrhythmias was significantly reduced to nearly wild-type levels (Figure 7F) in PolyQ-72 hearts overexpressing SOD compared to hearts expressing PolyQ-72 alone. Analysis of the myofibrillar organization also showed a partial rescue of PolyQ-72–induced myofibril disarray upon SOD-1 expression and both the size and the density of mutant PolyQ aggregates were markedly reduced (Figure 7G, 7H, 7K, 7L). Co-expression of SOD-2 and PolyQ-72 produced a similar suppression of the PolyQ-72-induced cardiomyopathy (Figure S5A to S5F). No cardiac defects were seen when SOD-1 or SOD-2 was co-overexpressed with the non-disease causing PolyQ-25 (not shown).

Over-expression of SOD has been shown to suppress the cardiac defects associated with knock-down of mitochondrial assembly regulatory factor (MARF) [35]. We attempted to rescue Poly-Q associated cardiac abnormalities with transgenic expression of MARF. However, over-expression of MARF does not result in any significant suppression of cardiac defects associated with mutant PolyQ-72 (Figure S5A to S5F). In fact, it has been shown that over-expression of mitofusion 2 promotes cardiomyocyte apoptosis via a mitochondrial death pathway in cultured mammalian cardiomyocytes [36].

We also tested the effects of feeding flies the antioxidant resveratrol. As for SOD-over-expression in PolyQ-72 hearts, resveratrol treatment reduced the dilated systolic and diastolic diameters, the diastolic and systolic intervals, and the arrhythmias (Figure 7A, 7B, 7F). It increased contractility and significantly reduced the PolyQ-72-induced increase in aggregate size and density (Figure 7C, 7I, 7K, 7L). These data indicate that the cardiac defects seen in response to expression of disease-causing PolyQ can be partially suppressed upon over-expression of antioxidant agents, such as SOD or resveratrol.

**Figure 8:**
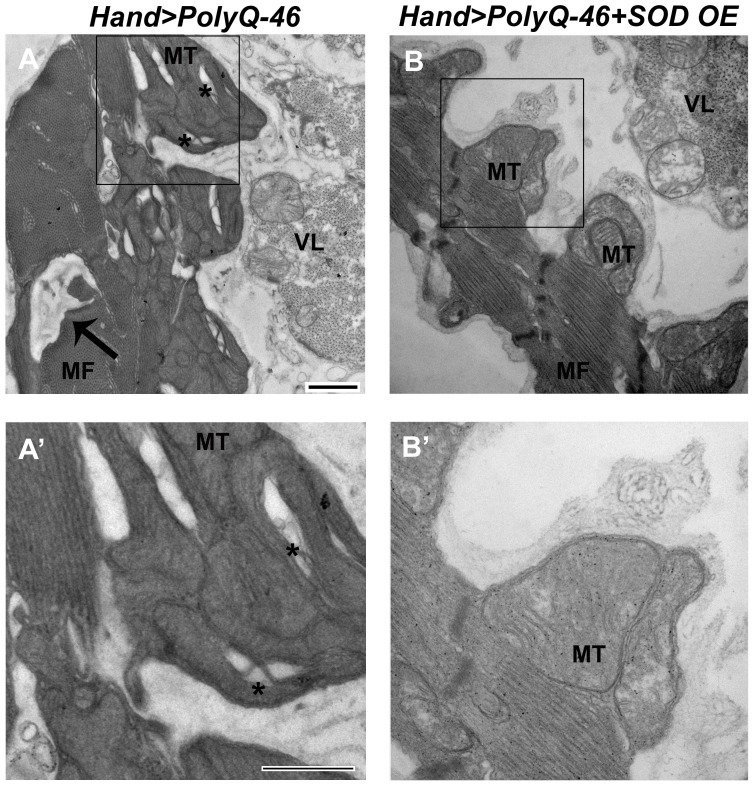
Mitochondrial ultrastructural defects in mutant Poly-Q expressing hearts are suppressed with SOD over-expression. (A, A') Cardiac expression of mutant PolyQ-46 resulted in myofibrillar degeneration (arrow), along with mitochondrial fragmentation (asterisks) in cardiomyocytes. (B, B') Over-expression of SOD in PolyQ-46 expressing hearts improved mitochondrial ultrastructure. MT indicates normally shaped mitochondria with densely packed cristae. Myofibrillar (arrow) degeneration was reduced to some extent in SOD-overexpressing hearts. MF refers to myofibrils. VL refers to non-cardiac ventral-longitudinal fibers. Scale bar is 500 nm.

Since over-expression of SOD or feeding with the antioxidant resveratrol rescued mutant PolyQ-induced cardiac defects, we examined whether over-expression of SOD could rescue the mitochondrial defects associated with expression of mutant PolyQ-46. In contrast to 4 week-old PolyQ-46 hearts, which contained areas of myofibrillar degeneration (Figure 8A, arrow), the majority of myofibrils in PolyQ-46 hearts overexpressing SOD were intact (Figure 8B, arrow). Hearts expressing PolyQ-46 contained fragmented mitochondria (Figure 8A, A' asterisks), while PolyQ-46 hearts overexpressing SOD contained normally shaped mitochondria with densely packed cristae (Figure 8 B, B'), similar to PolyQ-25 controls (Figure 4).

### Over-expression of the chaperone UNC-45 suppresses Poly-Q induced cardiomyopathy

It is known that accumulation of mutant PolyQ interferes with the protein folding machinery in neurons and it has been predicted that PolyQ has the same effect in the heart [37], [38]. Therefore, we reasoned that over-expression of the chaperone UNC-45, which may enhance proper protein folding, might improve cardiac function in hearts compromised by disease-causing PolyQ expression. To address this we over-expressed UNC-45 along with PolyQ-72 in the fly heart. Indeed, transgenic over-expression of UNC-45 completely suppressed mutant PolyQ-72 induced cardiac dilation (compare to *Hand*/+ wild-type controls, Figure 7A, 7B). UNC-45 over-expression improved contractility (Figure 7C) and the regularity of the heart rhythm (Figure 7F). Most interestingly, over-expression of UNC-45 in the presence of PolyQ-72 significantly reduced the density of mutant PolyQ aggregates compared to hearts expressing PolyQ-72 alone (Figure 7G, 7J, 7L, 7M, compare Figure 7G with 7J and compare lane 1 and 4 in Figure 7M). However, the mean aggregate size was not altered (Figure 7K). Additionally, hearts overexpressing UNC-45 showed a slightly more wild-type organization of actin-containing myofibrils (Figure 7J). Over-expression of UNC-45 in the presence of PolyQ-72 also resulted in a restoration some of the normal structure and content of the myosin-containing myofibrillar network (Figure S6B), which were nearly absent in the cardiomyocytes upon expression of PolyQ-72 alone (Figure S6A). These results suggest that disease-causing PolyQ may act by interfering with chaperone function, which is required for proper myosin folding/accumulation [28], [39]. In contrast, over-expression of UNC-45 alone or with PolyQ-25 resulted in only minor changes in functional cardiac parameters (Figure S7A to S7F). These results suggest that protein unfolding may play a role in mediating PolyQ-induced cardiomyopathy.

### Combined UNC-45 chaperone and antioxidant treatment is required for efficient suppression of PolyQ-induced aggregation and cardiac defects

**Figure 9:**
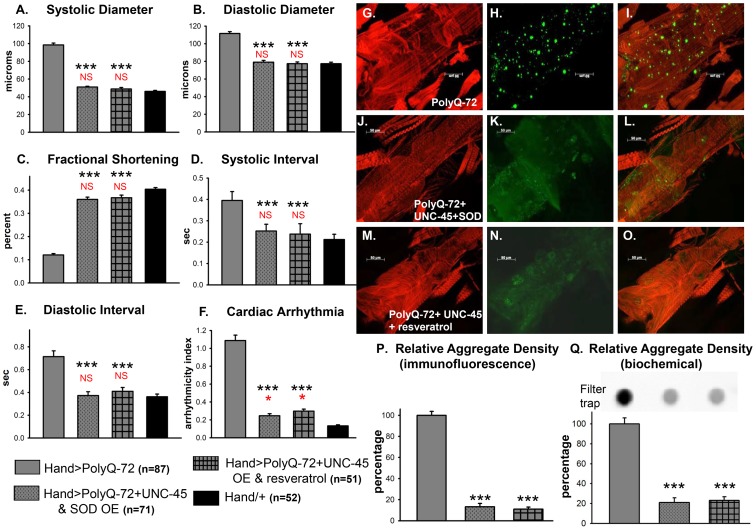
Combined over-expression of UNC-45 and SOD-1 or over-expression of UNC-45 and treatment with antioxidant resveratrol are required for nearly complete suppression of PolyQ-induced cardiac defects and aggregation. (**A**) Systolic diameters and (**B**) diastolic diameters of hearts from 3-week old flies expressing PolyQ-72 and either over-expressing both UNC-45 and SOD-1 or over-expressing UNC-45 with resveratrol treatment. Both treatments reduce cardiac dilation to wild-type values. (**C**) % FS is increased in response to both sets of manipulations to near wild-type levels. (**D**) Systolic intervals and (**E**) diastolic intervals of hearts from 3-week old flies; both treatments reverse the prolonged systolic and diastolic intervals observed in response to PolyQ-72 expression alone. (**F**) Cardiac arrhythmias in response to PolyQ-72 expression are reduced by both treatments to wild-type levels. Data shown as mean values ± SE; statistical significance was determined using one-way ANOVA and Dunnett's post hoc test. Significant differences were assumed for p<0.05. (*** = p<0.001). Red * and NS indicate differences relative to the *Hand*/+ control; black text indicates differences relative to mutant PolyQ-72. (**G–O**) Micrographs of hearts from 3-week old flies stained to show F-actin (phalloidin, red). (**G, J, M**) Hearts showed more organized myofibrils when co-overexpressing UNC-45 and SOD (compare G and J) or overexpressing UNC-45 and being treated with resveratrol (compare G and M). (**H, K, N**) PolyQ-72-induced GFP-tagged aggregates (green) were significantly reduced with both treatments (compare K and N to H). (**I, L, O**) Merged images. (**P**) Quantification of relative GFP-aggregate density (number of aggregates/unit area). Averaged data from 4 to 6 hearts is expressed as a percent relative to the mutant PolyQ. Over-expression of UNC-45 and SOD or over-expression of UNC-45 in the presence of resveratrol reduced the number of PolyQ-induced aggregates significantly. (**Q**) Quantification of relative GFP-aggregate content with a filter trap assay. Filter trap spot densities are shown at top, with densitometry scan results from averages of duplicate samples shown below; data are expressed as a percent relative to the mutant PolyQ. Compared to Htt-PolyQ-72, both over-expression of UNC-45 with SOD and over-expression of UNC-45 in the presence of resveratrol reduced PolyQ-induced aggregation significantly.

We examined whether improving protein folding and oxidative stress pathways might interact to suppress PolyQ-induced cardiac defects. To test this, we co-expressed UNC-45 and SOD-1 in conjunction with PolyQ-72. Co-expression of UNC-45 and SOD-1 restored cardiac contractility (Figure 9C) and suppressed cardiac dilation (Figure 9A, 9B), as well as cardiac arrhythmias (Figure 9F, and Movie S2). Over-expression of UNC-45 and SOD-1 also nearly completely suppressed the formation of GFP-positive aggregates that were dramatically induced by expression of PolyQ-72 (Figure 9H, 9K, 9P, 9Q). Furthermore, PolyQ-72 hearts expressing both UNC-45 and SOD-1 exhibited more organized actin-containing myofibrillar structures (Figure 9G vs. 9J). Together this suggests that mutant PolyQ aggregates induced by abnormal protein folding and increased oxidative stress are linked to cardiac physiological and structural defects.

To further confirm an association of both protein-unfolding and oxidative stress pathways with PolyQ-induced cardiomyopathy, we co-expressed PolyQ-72 and UNC-45 in the presence of the antioxidant resveratrol (Figure 9). Over-expression of UNC-45 in the presence of resveratrol almost completely suppressed PolyQ-induced cardiac dilation, cardiac arrhythmia and amyloid aggregation (Figure 9A, 9B, 9F, 9N, 9P, 9Q). Furthermore, the cardiac contractility was improved as was myofibrillar organization compared to PolyQ-72 expression alone (Figure 9C, 9M). Over-expression of UNC-45 and SOD or over-expression of UNC-45 in the presence of resveratrol also reduced the diastolic and systolic intervals to wild-type levels compared to age-matched PolyQ-72 (Figure 9D, 9E). A summary of cardiac parameters for wild-type controls as well as hearts expressing PolyQ-25 and PolyQ-72 (with or without antioxidant treatment) is shown in Figure S8A. Finally, mutant PolyQ-induced lifespan reduction was rescued by transgenic over-expression of SOD or co-expression of SOD and UNC-45 but not with UNC-45 over-expression (Figure S8B and Table S1). Overall, the genetic interactions that we have identified in this study demonstrate that the protein-misfolding and oxidative stress pathways induced by accumulation of HD-causing PolyQ aggregates are linked and associated with cardiac dysfunction.

## Discussion

Huntingtin protein is expressed in many tissues including the heart and epidemiological studies suggest that HD patients have a higher susceptibility to cardiac failure compared to age-matched controls without HD [8], [9], [11], [16], [17]. However, the cellular mechanisms underlying the cardiac dysfunction in HD have yet to be studied in the heart. Using the genetically tractable model system *Drosophila*, we now show a direct correlation between the levels of amyloid accumulation, overall ROS production and the severity of cardiac dysfunction. Cardiac-specific expression of disease-causing Htt-PolyQ (*PolyQ-46, PolyQ-72 and PolyQ-102*) all elicited cardiac dysfunction compared to hearts expressing the non-disease-causing PolyQ-25. In addition the qualitative as well as quantitative defects that we observed in response to PolyQ expression were dose-dependent. Since mutant Htt-PolyQ protein was expressed specifically in the heart, it is unlikely that our observations reflect a neuronal contribution to these cardiac defects. Our data suggest that the increased risk of cardiac disease in HD patients is possibly due to cardiac amyloid accumulation, mitochondrial defects as well as oxidative stress and that the severity of disease depends upon the length of the PolyQ repeat (Figures 3–5).

Our data also demonstrate that the likely cause of the observed functional defects is the severe myofibrillar disorganization and reduced myosin and actin content in myocardial cells resulting from cardiac-specific expression of disease causing PolyQ (Figure 3C and 3F). Recently we showed that the chaperone UNC-45 is required for preserving myosin accumulation/folding in *Drosophila* cardiomyocytes, as its reduction leads to severe disorganization of myosin-actin containing myofibrils and thus sarcomeres [28]. The current results extend this observation and are the first demonstration of a role for UNC-45 in amyloidosis-induced cardiac defects. In support of this hypothesis, it has previously been shown that nuclear or cytoplasmic aggregates (inclusion bodies) of polyglutamine proteins contain chaperones involved in protein folding [11], [37]. Furthermore, and consistent with our results, over-expression of the chaperone αB-crystallin reduces PolyQ-induced aggregation in rat neonatal cardiomyocytes; however, over-expression of αB-crystallin enhances amyloid oligomer formation and toxicity [23]. In the present study co-over-expression of UNC-45 with disease-causing PolyQ-72 dramatically reduced amyloid aggregate density (Figure 7L and 7M) and ameliorated cardiac dysfunction by decreasing the incidence of cardiac arrhythmia, suppressing the mutant-Htt-induced cardiac dilation (Figure 7A, 7B) and improving cardiac contractility to a dramatic extent (Figure 7C). Importantly, over-expression of UNC-45 in the presence of PolyQ-72 restored myosin-containing myofibrils (Figure S6), suggesting that one effect of amyloid aggregation is to interfere with proper folding of muscle myosin in cardiomyocytes.

The fact that UNC-45 over-expression did not completely suppress the mutant Htt-PolyQ-induced cardiac physiological defects and lifespan reduction is consistent with the idea that amyloid accumulation affects additional cellular pathways that result in cardiac abnormalities. As reported for αB-crystallin, suppression of aggregates is not sufficient to reduce toxicity [23] and this possibility may also exist in the case of UNC-45. It is also possible that the overall high level of oxidative stress produced by mutant PolyQ is the main determinant for lethality. Expression of mutant PolyQ leads to mitochondrial defects due to increased oxidative stress [4]–[10], [40]–[42]. Several neuronal studies have shown that expression of mutant polyQ affects SOD expression [43]–[46]. Manipulation of SOD seems to be directly correlated with levels of oxidative stress in several neurodegenerative diseases [4]–[10], [40]–[42]. Additionally, SOD over-expression reduces diabetic cardiomyopathy and some forms of neurodegeneration by reducing oxidative stress [47], [48]. However, neither UNC-45 nor SOD has been shown previously to suppress the PolyQ-induced phenotypes in either neuronal or cardiac animal disease models. Our data also support a role for oxidative stress pathways in amyloid-induced cardiac dysfunction and lethality. Treatment with oxidants aggravated the moderate effects of PolyQ-46 on heart function, causing an increase in amyloid aggregate density and more severe cardiac defects (Figure 6). This suggests a possibly causal relationship between oxidative stress, the formation of aggregates and cardiac dysfunction. Furthermore, our ultrastructural analysis clearly shows mutant PolyQ-induced mitochondrial defects, while DHE staining indicates that excess ROS production occurs upon expression of mutant PolyQ (Figures 4 and 5). Interestingly, some of the PolyQ aggregates co-localize with concentrated DHE staining (Figure 5). Significantly, we were able to reduce the size and density of mutant PolyQ-aggregates as well as the severity of the PolyQ-72-induced cardiac defects by over-expression of SOD or by feeding the anti-oxidant resveratrol (Figure 7). This is consistent with findings that resveratrol provides protection in neuronal models of Huntington's disease [49]–[54]. Interestingly, the anti-oxidant resveratrol has been shown to affect expression of anti-oxidative enzymes, including enhanced expression of SOD-1 [49]–[54].

Expression of mutant PolyQ may both induce oxidative stress and interfere with protein folding pathways [4], [21], [40]–[42]. A study using cultured mouse neurons showed that oxidative stress increases PolyQ aggregation and that over-expression of SOD1 in conjunction with the chaperone HSP-70/HSP-40 could suppress Htt-polyQ-induced aggregation and toxicity [42]. However, simultaneous manipulation of both of these genetic pathways has not previously been attempted *in vivo*. In addition to neurons, expression of the mutated Htt protein or expression of pre-amyloid oligomers cause cardiac defects by affecting several pathways including oxidative stress, mitochondrial abnormalities, presence of protein aggregates and increased autophagosomal content [10], [21], [30], [31], [34]. However, no attempt had thus far been made to suppress PolyQ-induced cardiac defects, a crucial step for understanding the mechanistic basis of disease progression and amelioration. Indeed, in our *in vivo* cardiac model, co-expression of UNC-45 and SOD-1 or expression of UNC-45 in the presence of resveratrol had a tendency to suppress the PolyQ-72-induced amyloid aggregation and concomitant cardiac dilation more efficiently than either treatment alone (Figures 7 and 9). Thus, our results suggest that suppression of both protein aggregates and ROS may be required for the amelioration of PolyQ-induced cardiomyopathy. As HD is primarily a neurological disease, the effect of such suppression is worth exploring in neural tissues.

In addition to interfering with protein folding pathways, expression of mutant PolyQ may lead to myofibril loss by directly interacting with muscle proteins. Previous studies have suggested that mutant PolyQ may bind directly to contractile proteins and disturb their function [55], [56]. Integrity of contractile proteins is also required for maintaining mitochondrial organization and cardiomyocyte function [35], [57]–[60]. Additionally, expression of aggregation-prone mutant PolyQ may induce oxidative stress due to mitochondrial damage in the cardiomyocytes, which are heavily dependent on mitochondrial function and are vulnerable to oxidative stress [60]–[62]. For example, knockdown of SOD results in mitochondrial defects and severe dilated cardiomyopathy phenotype in a mouse model [63]. Our results do show a dramatic increase in overall ROS levels in mutant PolyQ expressing hearts. Moreover, the GFP-positive PolyQ aggregates co-localize with areas of strong DHE staining and the observation that antioxidant treatments partially rescue the cardiac defects further support this hypothesis.

Overall, accumulation of amyloid in the cardiomyocytes can induce mechanical deficits by affecting the integrity of contractile proteins as well as mitochondria and lead to cardiomyocyte death, possibly through activation of autophagy. Consistent with our finding, a similar mechanism has been proposed for cardiomyopathy associated with amyloid producing mutant αB-crystallin [58]–[60], [64]. Both mutant αB-crystallin and mutant PolyQ caused aggregate formation in cardiomyocytes suggesting a common mechanism for underlying cardiomyocyte degeneration [21], [23], [58]–[60], [64]. It is unclear at this point whether the presence of toxic aggregates in cardiomyocytes is directly interfering with mitochondrial organization leading to cardiac defects or whether oxidative stress produced by mutant PolyQ leads to mitochondrial dysfunction that triggers cardiomyocyte dysfunction.

A full understanding of all the molecular details involved in mutant PolyQ induced cardiomyopathy will require additional study but we have now identified some of the key players and interactions *in vivo*. Although dense granular deposits, immunoreactive to an anti-Huntingtin antibody, have been found in muscle tissue from an HD patient, no such study has been performed on HD heart biopsy samples [65]. Thus, our study suggests that it would be useful to look for accumulation of amyloid protein in the hearts of HD patients, especially those with heart disease. Delineating how these aggregates might be toxic to cells will be critical not only for an understanding of PolyQ-induced cardiomyopathy but also for gaining insights into aggregation-based neural degeneration. The *Drosophila* heart model provides a genetically tractable system whereby these interactions can be examined in the context of a functioning organ. Indeed, elucidating the genetics underlying PolyQ-induced cardiomyopathy should also have an impact on our understanding of other cardiac diseases associated with oxidative stress, mitochondrial dysfunction, the unfolded protein response and proteostasis in general.

## Methods

### *Drosophila* stocks and screening system for PolyQ-induced cardiomyopathy

To evaluate cardiac function following PolyQ-induced cardiomyopathy, we obtained *Drosophila* transgenic lines expressing enhanced-GFP-tagged mutant Htt fragments (UAS-Httex1-QneGFP) with different PolyQ lengths (Q25, Q46, Q72, and Q102) [5]. Heart-specific expression was achieved with a UAS-Gal4 system using the Hand driver [66], [67], crossed to the different UAS-Httex-GFP lines (*Httex1-Q25-eGFP*, *Httex1-Q46-eGFP*, *Httex1-Q72-eGFP* and *Httex1-Q102-eGFP*). For simplicity *Httex1-Q25-eGFP*, *Httex1-Q46-eGFP*, *Httex1-Q72-eGFP* and *Httex1-Q102-eGFP* are referred to as *PolyQ-25, PolyQ-46, PolyQ-72 and PolyQ-102*, respectively in this study. F-1 progeny (*i.e. Hand-Gal4/+, Hand-Gal4>UAS-PolyQ-25, Hand-Gal4>UAS-PolyQ-46, Hand-Gal4>UAS-PolyQ-72* or *Hand-Gal4>UAS-PolyQ-102*) of each transgenic cross were collected, separated by sex and cultured at 25°C. Lifespan of female progeny was determined with survivorship being monitored every third day with a food change as previously described [28]. Adult flies were analyzed at 1 and 3 weeks of age. The cardiac tissue-specific *Hand*-Gal4 driver was gift from Eric Olsen [68]. Transgenic *unc-45*, *SOD* and *MARF* lines were generated as previously described [69]–[71].

### Semi-intact *Drosophila* heart preparation cardiac function analysis

Semi-intact hearts were prepared as described previously [29], [72]. Direct immersion optics were used in conjunction with a digital high-speed camera (up to 200 frame/sec, Hamamatsu EM-CCD) to record 30 s movies of beating hearts; images were captured using HC Image (Hamamatsu Corp.). Cardiac function was analyzed from the high speed movies using semi-automatic optical heartbeat analysis software (a MatLab-based image analysis software) which quantifies heart period, diastolic and systolic diameters, diastolic and systolic intervals, cardiac rhythmicity, fractional shortening and produced the M-mode records [29], [72].

### Analysis of mutant Htt-induced structural defects

Dissected hearts (from 1 and 3 week old flies) were briefly exposed to 10 mM EGTA and then fixed with 4% paraformaldehyde in PBS as previously described [73]. Fixed hearts were probed with myosin antibody followed by goat-anti-rabbit IgG-Cy5 (Chemicon, Temecula, CA) and Alexa555-phalloidiin (Invitrogen, Carlsbad, CA) to stain F-actin. Fluorescence imaging of *Drosophila* heart tubes was carried out using an Apotome Imager Z1 (Zeiss) and an AxioCam MRm (Zeiss) as previously described [73], [74]. To detect extended PolyQ-induced aggregates in the fly heart, we used Htt-PolyQ-GFP [5] in conjunction with anti-myosin or phalloidin.

### DHE and LysoTracker staining

Dihydroethidium (DHE) and LysoTracker were employed for the detection of oxidative stress and autophagosomes/lysosomes respectively using a modified protocol previously described for use in other tissue [71], [75]. Briefly, semi-intact hearts were prepared as described above and stained with DHE (Molecular Probes, Carlsbad, CA) at 2 µM final concentrations in artificial hemolymph for 30 min, followed by three washes with artificial hemolymph. Hearts were relaxed with 10 mM EGTA and mounted in Vectashield. A similar staining procedure was applied for the staining with LysoTracker red (Molecular Probes, Carlsbad, CA): 30 min, 1 µM final concentration in artificial hemolymph. Fluorescence imaging was carried out using an Apotome Imager Z1 (Zeiss) and an AxioCam MRm (Zeiss) as previously described [73], [74]. DHE intensity was quantified using ImageJ software.

### Ultrastructural analysis of mutant-Htt induced cardiac defects

Semi-intact heart preparations were prepared for transmission electron microscopy using a modified protocol described previously [76]. Briefly, hearts were relaxed with 10 mM EGTA followed by a primary fixation protocol (3% formaldehyde, 3% glutaraldehyde in 0.1 M cacodylate buffer, pH 7.4) and secondary fixation (1% OsO_4_, 100 mM phosphate buffer, and 10 mM MgCl_2_, pH 7.4). The samples were block stained in 2% uranyl acetate and dehydrated with an acetone series, followed by orientation and embedding in Epon-filled BEEM capsules. Polymerization was performed at 60°C under vacuum. Thin sections (50 nm) were cut using a Diatome diamond knife on a Leica ultramicrotome and picked up on formvar-coated grids. Slices were stained with 2% uranyl acetate for 10 min and Sato's lead stain [77] for 2 min. Images were obtained at 120 kV on a FEI Tecnai 12 transmission electron microscope.

### Transgenic suppression of Poly-Q induced hearts

We employed standard genetic and transgenic techniques [69] to co-express chaperones or SOD in flies expressing UAS-PolyQ in the heart using the Gal4 driver. Genetic crosses using multiple balancers were carried out with transgenic flies expressing *unc-45*, *SOD-1* or *SOD-2*. Briefly, the wild-type genomic d*unc-45* was used as previously described [69]. To ameliorate PolyQ-72 induced cardiac defects, adult males homozygous for the PolyQ-72 (*w^1118^*/Y; *+/+*; *PolyQ-72*/*PolyQ-72*, flies were crossed with female flies homozygous for the wild-type d*unc-45* transgene on the 1st chromosome *P[w^+^*, d*unc-45/P[w^+^*, d*unc-45]; Hand-Gal4/Cyo; +/+* [69]. The following progeny (*P[w^+^*, d*unc-45]/w1118; Hand-Gal4/+; PolyQ-72/+*) were analyzed to determine ability of UNC-45 over-expression to suppress Poly-Q induced cardiac defects. A similar genetic suppression approach was used with the wild-type d*unc-45* transgene on the 2nd chromosome after crossing *w^1118^/w^1118^*; *P[w^+^*, d*unc-45]*/*P[w^+^*, d*unc-45]*; *+/+* to *w^1118^*/Y; *Cyo/Hand-Gal4*; *PolyQ-72*/*PolyQ-72* to obtain *w^1118^/w^1118^*; *P[w^+^*, d*unc-45]*/*Hand*; *+/PolyQ-72*. For expression of *SOD-1* (2^nd^ chromosome), *w* ^1118^ */w^1118^*; *P[w^+^*, *SOD-1]*/*P[w^+^*, *SOD-1]*; +/+ flies were crossed to *w^1118^*/Y; *Cyo/Hand-Gal4*; *PolyQ-72*/*PolyQ-72* to obtain *w^1118^/w^1118^*; *P[w^+^*, *SOD-1*]*/Hand*; +/PolyQ-72. Finally, for co-expression of *unc-45*, *SOD-1* in PolyQ-72 expressing flies, females homozygous for the wild-type d*unc-45* transgene on the 1st chromosome *P[w^+^*, d*unc-45]/P[w^+^*, d*unc-45]; Hand-Gal4/Cyo; +/+ were crossed to w^1118^/Y*; *P[w^+^*, *SOD-1]*/Cyo; *PolyQ-72*/*PolyQ-72* to obtain *P[w^+^*, d*unc-45]/w^1118^; Hand-Gal4/SOD-1; PolyQ-72/+*. Similar genetic approaches were used to co-express UNC-45 or SOD in flies expressing UAS-Poly-25 in the heart using the Gal4 driver. To study the effects of MARF over-expression on mutant PolyQ-induce cardiac defects, flies with MARF overexpressing transgenes on their second or third chromosomes were crossed with extant stocks (*w^1118^*/Y; *Cyo/Hand-Gal4*; *PolyQ-72*/*PolyQ-72*) to obtain *w^1118^/w^1118^*; *P[w^+^*, *MARF]/Hand; +/PolyQ-72* or *w^1118^/w^1118^*; *+/Hand; P[w^+^, MARF]/PolyQ-72*. We did not see any difference in the time to eclosion or the number of progeny between the mutant PolyQ and cardiac-specific SOD1/UNC-45/MARF overexpression lines.

### Pharmacological manipulations and hydrogen peroxide treatment

For treatment with resveratrol, flies expressing *Hand-Gal4>UAS-PolyQ* (control or disease causing), *Hand*-Gal4; *w* ^1118^ or (*P[w^+^*, d*unc-45]/w^1118^; Hand-Gal4/+; PolyQ-72/+*) were raised separately in standard media in the presence or absence of resveratrol (final concentration, 1 mg/ml, a dose previously used in the *Drosophila* model [48]). Flies were collected on eclosion, separated by sex and cultured at 25°C; food was changed every 3 days. Adult flies were analyzed at 3 weeks of age. Additionally, we did not see any difference in the time to eclosion or the number of progeny between the mutant PolyQ and resveratrol fed organisms (concentration 1 mg/ml). For treatment with H_2_O_2_, flies were collected on eclosion and separated by sex. One group was cultured on food containing 1% H_2_O_2_ and a second group received standard food. Food was changed every 3 days and adult flies were analyzed at 3 weeks of age.

### Quantification of amyloid-aggregates from immunofluorescence micrographs

Semi-intact hearts from flies expressing various length Htt-PolyQ-GFP [5] were prepared and fixation was carried out at described above. The numbers of aggregates in micrographs taken using the GFP wavelength (488 nm) were quantified using ImageJ software (Particle Count and Analysis function). Briefly, each immunofluorescence micrograph was divided into 50×50 µM square boxes (2500 µm^2^ unit area) and the total number of aggregates within each box was quantified. Between three and four boxes per heart were analyzed and the number of aggregates per box was averaged for each heart. We also quantified the 2D surface area of the aggregates within the box using ImageJ software. Thus, we quantified mutant Htt-polyQ deposits in terms of the total number of aggregates per unit area as well as in terms of the size of aggregates from at least four hearts per genotype. For each heart aggregate density/size was determined for each of three 50×50 micron regions and averaged and four to six hearts were examined for each genotype/treatment.

### Biochemical assay for the detection of protein aggregates

We used a filter trap assay for the quantification of Htt-polyQ GFP aggregates in the *Drosophila* heart as previously described [30]–[32]. Briefly, hearts were dissected (30–50/genotype) and harvested in SDS lysis buffer (20 mM Tris-HCl, pH 7.5, 200 mM NaCl, 2% SDS); samples were diluted to 1 µg/100 µl with TBS (20 mM Tris-HCl, pH 7.5, 500 mM NaCl) and 500 ng were loaded onto a cellulose acetate membrane (0.2 µm pore size, Whatman, Piscataway, NJ), using the 96-well BioDot Apparatus (Bio-Rad, Hercules, CA). Protein content of homogenized heart samples was determined using the DC Protein Assay Kit II (Bio-Rad, Hercules, CA). Non-specific binding sites were blocked using 5% nonfat milk in TBA buffer for 2 hours. Immunodetection was performed after incubation with mouse anti-GFP (Covance Research Products, Dedham, MA) and secondary antibodies (KPL, Inc., Gaithersburg, MD) using Thermo Scientific SuperSignal West Dura Substrate on a Bio-Rad ChemiDoc XRS System. A parallel series of 500 ng samples was blotted onto nitrocellulose (Bio-Rad, Hercules, CA) to confirm GFP expression in non-aggregating PolyQ-GFP, using H2B antibody (Cell Signaling, Danvers, MA) as a loading control. Quantification of the immunoblot's density was carried out using ImageJ software.

### Statistical analysis

For all quantitation except lifespan analysis, statistical significance was determined using one-way analysis of variance (ANOVA) followed by Dunnett's post-hoc test to determine significance between groups with Prism 6.0 (Graph Pad) software. Significant differences were assumed for p<0.05. For lifespan studies, data were analyzed using the Gehan-Breslow-Wicoxon test followed by multiple comparisons between control and experimental groups. Significance was taken at p values less than the Bonferroni-corrected threshold of p<0.0125.

## Supporting Information

Figure S1 Physiological cardiac defects associated with mutant Poly-Q in 1-week old flies. (A) Systolic and (B) diastolic diameters of the hearts from PolyQ-46, PolyQ-72 and PolyQ-102 expressing flies were significantly higher than those of age-matched controls (*Hand/+* or PolyQ-25). Cardiac contractility was quantified as % fractional shortening (C) in the hearts expressing PolyQ-46, PolyQ-72 and PolyQ-102, which showed significantly reduced contractility compared to control hearts. Hearts expressing *PolyQ-46*, *PolyQ-72* and *PolyQ-102* show prolonged systolic (D) and diastolic (E) intervals compared to control hearts (*Hand*/+ or *PolyQ-25*). (F) Cardiac arrhythmia was significantly increased in hearts expressing *PolyQ-46*, *PolyQ-72* and *PolyQ-102* compared to controls. All data are shown as mean ± SE; statistical significance was determined using one-way ANOVA and Dunnett's post-hoc test; *p<0.05, **p<0.01, ***p<0.001.
(EPS)

Figure S2 Mutant PolyQ induces autophagy. (A–C) Immunofluorescence micrographs showing GFP fluorescence and LysoTracker staining in a heart expressing PolyQ-25 GFP. This control heart shows very little GFP or LysoTracker signal. (D–F) GFP fluorescence and LysoTracker staining in a heart expressing PolyQ-72 GFP. Expression of mutant PolyQ-72 results in the dramatic appearance of LysoTracker positive punctae; the majority of them co-localize with GFP positive aggregates. Scale bar is 50 µm.
(EPS)

Figure S3 Oxidative stress alone results in mild cardiac dysfunction but without cardiac dilation and myofibril loss. (A) Systolic and (B) diastolic heart diameters are not altered by H_2_O_2_ feeding in wild-type (*Hand*/+) or control PolyQ-25 expressing hearts. (C) % FS was slightly reduced by feeding oxidant in both wild type and PolyQ-25 controls. (D) Diastolic and (E) systolic intervals were moderately prolonged in both wild type and PolyQ-25 expressing controls upon feeding H_2_O_2_. (F) Cardiac arrhythmia was slightly increased in the presence of oxidant compared to age-matched *Hand*/+ and PolyQ-25 expressing hearts without oxidant. Hearts were from 3-week old flies; data are shown as mean ± SE; statistical significance was determined using one-way ANOVA and Dunnett's post-hoc test; *p<0.05, **p<0.01, ***p<0.001). (G and H) Hearts from 3-week old *Hand*/+ flies show actin-containing myofibrils (red) within the cardiomyocytes without oxidant (G) and with oxidant (H). (I and J) Hearts from 3-week old flies expressing *PolyQ-25* show actin-containing myofibrils (red) and GFP (green) within the cardiomyocytes without (I) and with oxidant (J).
(EPS)

Figure S4 Oxidative stress aggravates the effects of PolyQ-72 on cardiac function. (A) Cardiac arrhythmias (quantified as the Arrhythmicity Index) were significantly increased upon feeding of the oxidant H_2_O_2_ (3-week old flies). (B) Feeding flies the oxidant H_2_O_2_ results in a further reduction of cardiac contractility (% FS) compared to cardiac specific expression of PolyQ-72 alone. Data are shown as means ± SE; statistical significance was determined using one-way ANOVA and Dunnett's post-hoc test; *p<0.05, **p<0.01, ***p<0.001.
(EPS)

Figure S5 Over-expression of SOD-2, but not MARF, rescues mutant Poly-Q induced cardiac dysfunction. (A) Systolic diameters and (B) diastolic diameters of hearts from 3 week old flies over-expressing PolyQ-72, or PolyQ-72 plus SOD-2 or MARF. Diameters are reduced toward wild-type values only by the SOD-2 over-expression (compare Figure S4A, S4B with Figure 2A, 2B). (C) The depressed cardiac contractility (% FS) of hearts expressing PolyQ-72 is restored upon over-expression of SOD-2, but remains depressed with MARF over-expression. (D, E) The PolyQ-72 induced increase in systolic and diastolic intervals (decreased rate) was restored toward wild-type levels by over-expression of SOD-2 but not MARF (compare Figure S5D, S5E with Figure 2D, 2E). (F) The increased cardiac arrhythmia in response to PolyQ-72 expression is reduced upon over-expression of SOD-2 but not with MARF over-expression. Data shown as mean ± SE; statistical significance was determined using one-way ANOVA and Dunnett's post-hoc test; *p<0.05, **p<0.01, ***p<0.001.
(EPS)

Figure S6 Over-expression of UNC-45 rescues myofibrillar myosin in hearts expressing PolyQ-72. (A) The circumferential myofibrillar organization in myocardial cells expressing PolyQ-72 is severely disrupted (thin arrows). Non-cardiac longitudinal fibers are still present and contain myosin (thick arrow). (B) Co-expression of UNC-45 with disease-causing PolyQ-72 results in a restoration of the myosin-containing circumferential myofibrils (thin arrows). Scale bar is 20 µM.
(EPS)

Figure S7 Impact of UNC-45 over-expression on cardiac performance in control flies. Over-expression of UNC-45 in both of the control lines used in this study (*Hand/+* and *Hand>PolyQ-25*) had no effect on (A) systolic diameters. (B) Diastolic diameters were slightly smaller in the hearts over expressing UNC-45 compared to *Hand*/+. (C) Cardiac contractility (% FS) of hearts over-expressing UNC-45 was slightly reduced compare to *Hand*/+ controls. (D, E) Over-expression of UNC-45 also resulted in a slight lengthening of the (D) systolic and (E) diastolic intervals compared to the *Hand*/+ control without UNC-45 (see also Figure 2D, 2E). (F) Cardiac arrhythmias in control hearts over-expressing UNC-45 were slightly increased compared to *Hand/+* controls. Data shown as mean ± SE. Statistical significance was determined using a one-way ANOVA and Dunnett's post hoc test; *p<0.05, **p<0.01, ***p<0.001, NS - no statistical difference.
(EPS)

Figure S8 Summary of the qualitative cardiac parameters and lifespan. (A) The percent of all hearts exhibiting defective ostia, one or more non-contractile regions, or non-beating hearts (complete asystole) is shown. All of the PolyQ-72 expressing hearts show some form of dysfunction compared to wild-type (*Hand*/+) and polyQ-25 controls. Over-expression of both UNC-45 and SOD reduced the incidence of these qualitative cardiac defects induced by PolyQ-72 expression. Similar results were obtained upon co-expression of PolyQ-72 and UNC-45 in the presence of resveratrol (not shown). (B) Mutant PolyQ-72 induced lethality was rescued by transgenic over-expression of SOD or co-expression of both SOD and UNC-45 (p<0.001). Over-expression of UNC-45 alone did not rescue the PolyQ-72 induced decrease in lifespan significantly. Graph plots % survival vs. days post-eclosion (n = 150–200 for each group).
(EPS)

Movie S1 Morphological and functional cardiac defects associated with mutant Poly-Q. PolyQ-25 (control, top), PolyQ-46 (middle) and PolyQ-72 (bottom) expressing hearts from 3-week old *Drosophila*. PolyQ-25 hearts typically show a regular beating pattern. PolyQ-72 expressing hearts exhibit significant dilation, arrhythmia and reduced contractility. The effects of PolyQ-46 expression are milder than those in response to PolyQ-72. Movies are 20 s long at 120–150 fps.
(MP4)

Movie S2 Suppression of mutant PolyQ-72 induced morphological and functional cardiac defects with UNC-45 and SOD over-expression. Heart from 3-week old flies expressing PolyQ-72 (top) and PolyQ-72 co-expressed with UNC-45 and SOD (bottom). UNC-45 and SOD completely rescue the PolyQ-72 induced dilation, arrhythmia and reduced contractility. Movies are 16 s long at 120–150 fps.
(MP4)

Table S1 Summary of lifespan statistics. Lifespan statistics of control and mutants groups as well as between mutant and suppressor groups. Data were analyzed using the Gehan-Breslow-Wicoxon test followed by multiple comparisons between control and experimental groups. Significance was taken at p values less than the Bonferroni-corrected threshold.
(EPS)
